# Simulating forage plantain growth and defoliation in APSIM^☆^

**DOI:** 10.1016/j.mex.2025.103530

**Published:** 2025-07-23

**Authors:** Rogerio Cichota, Xiumei Yang

**Affiliations:** The New Zealand Institute for Plant & Food Research Limited, Lincoln, New Zealand

**Keywords:** *Plantago lanceolata*, Grazing systems, Pasture, Mixed sward, Crop system modelling, New Zealand

## Abstract

The methodology used to develop and test a crop system model to simulate growth and defoliation management of plantain forage (*Plantago lanceolata* L.) in the Agricultural Production Systems Simulator (APSIM) framework is presented. The model has been primarily developed based on data from New Zealand, but it should be applicable in other regions, and it can be extended to account for new cultivars being released in the market. The model is capable of simulating pure forage plantain stands and can also be used in mixed swards with other forage species within the APSIM framework. Validation tests for the current stage focused on pure forage plantain stands in New Zealand; results showed a good model performance when predicting biomass accumulation over a growing season and nitrogen content (with NSE > 0.8), although predictions for individual defoliation events were not very accurate (NSE between -1.0 and 0.6). More data is needed to improve how the model describes biomass partition among plant organs and how this is affected by environmental conditions and defoliations. This model contributes to the ongoing efforts to explore alternative crops and improve management of grazing systems, aiming to reduce environmental impacts while maintaining productivity.

• Data to describe various aspects of plantain forage were gathered from literature and field trials
• Model built to simulate plant growth and re-growth after defoliations within APSIM
• The model can be used in monoculture and mixed swards

## Graphical abstract

**Figure:**
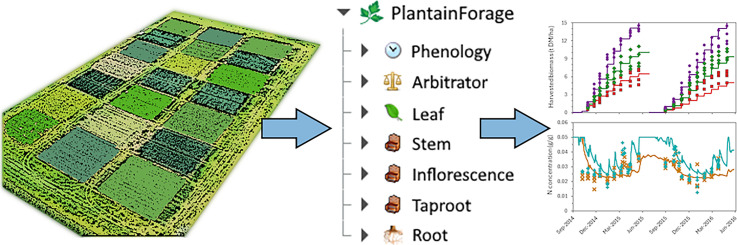

## Specifications table

**Table:** 

Subject area	Agricultural and Biological Sciences
More specific subject area	Crop growth modelling, forage production and animal feed, also related to Environmental Science, on water quality and greenhouse gases emission.
Name of your method	PlantainForage Model
Name and reference of original method	N/A
Resource availability	APSIM simulation model APSIM documentation Validation simulation

## Background

Plantain forage (*Plantago lanceolata* L.), also called ribwort plantain, is a herbaceous plant to the grasslands of Eurasia [1,2]. However, it is now common worldwide in areas with temperate and subtropical climates, where it is often considered a weed. Plantain varieties have been selected to be used as animal forage in New Zealand since the 90′s [2,3]. The plants are easy to establish, drought resistant, highly palatable, and with potential health benefits to grazing animals [[2], [3], [4]]. More recently, plantain has been promoted as a forage component in grazing systems due to its ability to reduce nitrogen (N) losses with minimal impacts on production [[5], [6], [7]]. There are a few potential mechanisms by which plantain can affect N cycling through the farm system. These include a diuretic effect [8,9], changes on N partitioning in ruminants [6,10], and the presence of compounds that act as biological nitrification inhibitors [11,12].

The plant consists of a rosette of basal semi-erect leaves which may produce one or more flowering stalks. The leaves have a characteristic lanceolate shape with 3 to 5 parallel veins along the length with few or no hairs. The reproductive stems, or stalks, are leafless, growing to about 10–15 cm above the top of the canopy and have scattered hairs near their base. Each stalk ends in an ovoid inflorescence, 1–4 cm long, containing many small flowers. The shape and colour of the inflorescence changes over time (from a cone to a cylinder) as the flowers mature from the base to the top. The flowering period starts in late spring and goes until early autumn, with considerable variations between plants of the same stand. Forage plantain is considered a long-day plant [13] and does not need vernalisation to trigger flowering. The flowers are wind-pollinated and when fertilised produce a small seed capsule containing two small seeds, with weight varying between 1.5 and 2.0 mg per seed [3,14]. Seeds have an oblong shape and their colour is dark brown to black. The root system consists of a relatively shallow crown of coarse fibrous roots, including adventitious roots, and several long lateral roots [15,16]. Although technically a taprooted species, plantain often does not have the dominant deep main root. Nonetheless, the thick roots are the primary storage organ [16,17]. Plantain forage can allocate a greater proportion of roots at depth, giving a competitive advantage over shallow-rooted grasses [2,18].

Selection of plantain cultivars for use as forage has focused mostly on narrow-leaf varieties, which have an erect, bushy, growth habit and the ability to tiller under grazing. More recently, some emphasis has also been placed on breeding for greater growth during cold periods [2,13]. Forage plantain can grow large leaves, with length up to 40 cm instead of typical 15–25 cm of wild varieties, and these are highly palatable to grazing animals. Conversely, reproductive organs are less palatable and mature stalks are avoided by grazers [19,20]. Plantain forage has high nutrition value, good digestibility, and its mineral content is usually higher than that of ryegrass-clover swards [2,[21], [22], [23]]. It also has a lower tissue dry matter content (or higher moisture content) compared with most grasses. This implies that animals have to ingest a greater amount of fresh biomass for the same dry-matter intake, which at least partially explains the dilution of urinary N [8,9]. Despite the high moisture content, it is possible to produce good quality silage from swards that include plantain [24].

While there is a growing body of evidence from New Zealand for the benefits of including plantain in swards and in the diet of ruminants [6,23], there are many questions on how to effectively use this forage in various farming systems and how to best manage it in different environments. Process-based modelling provides an excellent platform to synthesise current agronomic knowledge and to explore scenarios with varying management in different farming systems. Models can also help to provide quantitative assessments of the environmental impact of such systems. This is particularly important in New Zealand with regards to recent regulation requiring a reduction in N losses from grazed pastures to freshwater [23,25]. The objective of this work is to provide the details of a modelling tool that can simulate the growth and management of plantain forage within the APSIM (Agricultural Production Systems Simulator) Next Generation modelling framework [26] (see www.apsim.info). Developing the model within a process-based, systems framework, such as APSIM allowed us to focus the growth and development of the crop while enabling to simulate a variety of processes associated with or deriving from the use of forage plantain. These include water balance and N supply from the soil, biomass senescence or wastage interacting with soil organic matter, N losses via leaching or gas emissions, and competition with other species (although some of these need further development and validation). APSIM is also very flexible to capture management actions in detail, thus enabling setting up a variety of farming system and investigate alternative management practices. Although the model development focuses strongly on New Zealand farming systems, the APSIM model framework is general and can be used in other regions and systems. The model can also be easily extended in the future to include new cultivars. The methods presented here focus on describing the development and biomass production of pure plantain swards. The model can be used in mixed swards with other forages in APSIM, but its performance in such systems is the scope of ongoing work. Further development is needed for some potential uses of the model (e.g., estimate seed production) and to cover specific processes related to the interactions with animal physiology and soil transformations that are important in driving the documented effects of forage plantain on nitrogen losses.

## Method details

### Overview of model implementation

All development was made using APSIM Next Generation (release number 7650.0, Jan 2025, is the reference for this work). APSIM is an open-source modelling framework composed of several sub-models [26,27] that describe a range of processes in the soil and plants, and it can simulate a wide variety of farming systems (full description and documentation of the framework can be found on www.apsim.info; the code and test simulations can be accessed via github.com/APSIMInitiative/ApsimX). This work focuses on the description of the plant growth model and the setup of validation tests. The description and parameterisation of other sub-models (e.g. soil water balance) are given briefly and only where necessary.

**Fig. 1:**
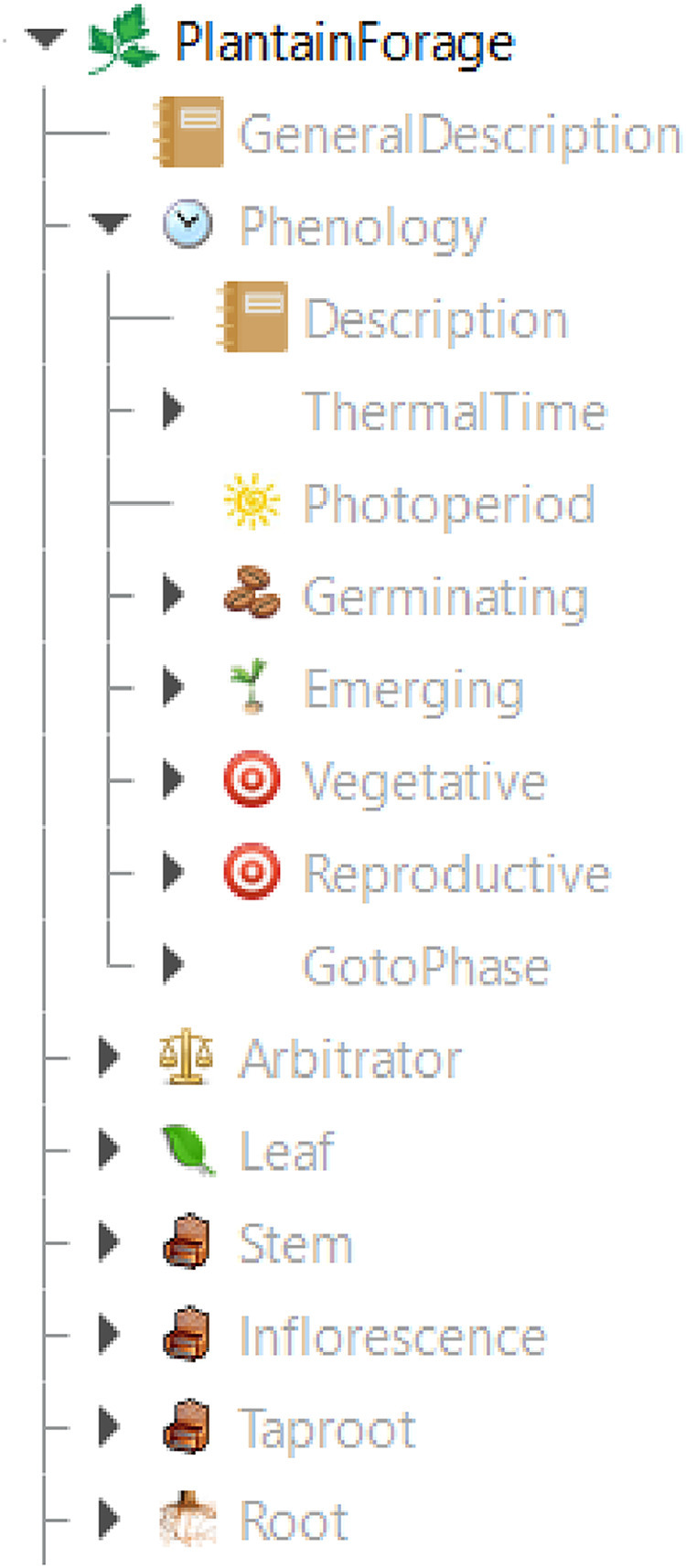
Basic schematics of the various phenological phases and organs that comprise the PlantainForage model. The progression between the four phenological phases (germinating, emerging, vegetative and reproductive) are controlled by ThermalTime and Photoperiod. The GotoPhase represents a reset to the vegetative phase at the end of the reproductive phase. The allocation of newly photosynthesised or re-translocated biomass (C and N) between the five organs (Leaf, Stem, Inflorescence, Taproot, and Roots) is controlled by the organ Arbitrator.

The PlantainForage model was built using the Plant Modelling Framework (PMF) [28], aiming to simulate the growth of a plantain forage crop, either as a monoculture or in multi-species swards. The model focuses primarily on describing plant growth from sowing to maturity and regrowth after defoliations. It considers only a simplified account of the reproductive phase, without an explicit consideration of flower development and seed production (these may be included in future releases). As with any model built using PMF, the PlantainForage model is composed of sub-models describing the various organs and phenology phases of plantain (Fig. 1). It is important to note that the model aims to describe the average behaviour of the plant population, without explicitly including population dynamics. This results in a somewhat fuzzy definition of the various development stages and the partition of biomass between the various organs. Similar to most forages, a population of plantain is likely to contain young and mature plants, as well as having some plants with mature seeds while others are just developing flower stalks. This provides further justification for a simplified reproductive phase where stalks, flowers, and seed are all developing simultaneously. Furthermore, the model describes a perennial crop, with phenology re-setting to the vegetative stage at the end of the reproductive phase. In reality, plantain persistence is usually observed to be limited in grazing pasture systems due to competition with other forages, with population declining sharply after about two-three years [29,30]. Given the abundance of seed production in plantain, some level of natural re-seeding is likely to occur [31]. However, in grass-dominant swards this appears to be insufficient to maintain a stable level of plantain in the sward [30,32], with farmers actively over- or under-sowing the swards regularly to compensate.

### Phenology

Both thermal time and photoperiod are used to simulate the progression through phenological phases of plantain. From sowing, the model uses a simple set of phases to account for germination and emergence stages, after that the model cycles between a vegetative and a reproductive phase. The switch between vegetative and reproductive phases is controlled by the accumulation of days with long sunlight [13,33,34], and the model assumes the reverse to end the reproductive phase, at which point the model resets to the vegetative phase.

**Fig. 2:**
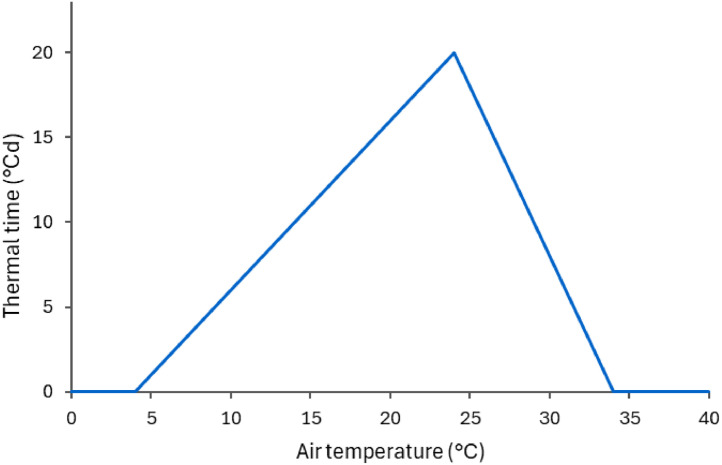
Daily thermal time computed as a function of the average air temperature used in the PlantainForage model.

The thermal time, or cumulative growing degrees days, is calculated from the daily average temperature using three cardinal temperatures: minimum, maximum, and optimum (Fig. 2). Crop development accelerates as temperature increases from minimum to optimum and then slows down, ceasing completely at maximum temperature. There is very little information about the temperature thresholds for plantain forage. The minimum temperature seems to be particularly variable, as one of the traits that varies among cultivars is winter activity [35]. Based on data for wild varieties as well as similar species (*Plantago asiatica*), it is possible to infer that the minimum temperature varies between 0 and 14 °C, while optimum temperature sits just above 20 °C [36,37]. Plantain forage is reported as tolerant to high temperatures [2] and will outgrow ryegrass over summer [20]; the available data suggest that maximum temperature is above 30, perhaps as high as 38 °C in some varieties [36,37]. Since these values are not specific for cultivars of forage plantain currently used in New Zealand, the uncertainty surrounding these parameters represents a major knowledge gap and hinders the use of models for identifying the best cultivars and management strategies for different locations.

Following the conventions of PMF, germination in the PlantainForage model is assumed to occur one day after sowing, provided that the soil moisture is greater than zero. Variations between stands are solely accounted for in the emergence phase, following germination. The model assumes a 100 % germination rate. Typical reported values for germination rates vary between 75 and 95 % [14,21], therefore the user must adjust the sowing rate to achieve the correct plant density. Plantain forage has small seeds and to ensure even emergence, it is recommended that sowing depth should be <10 mm [38,39]; seeds may remain dormant for years deeper in the soil. As variations in germination rate are not explicitly simulated by the PlantainForage model, the sowing depth will only affect timing of emergence in the current version. This is controlled by the elongation rate, which is assumed to be a function of thermal time (Eq. (1)). The elongation rate for seedlings was parameterised based on typical values for forage crops as no specific data are available for plantain.

(1) $$\tau_{t,emerg}=15+10d_{S}$$

where $\tau_{t,emerg}$ is the accumulated thermal time ( °Cd) required to complete the emergence phase and $d_{S}$ is the sowing depth (mm).

The vegetative phase begins immediately after the plants emerge from the ground and is also triggered after the end of the reproductive phase (phenology reset). During this phase, only the vegetative organs (roots, taproots, and leaves) are allowed to grow. A period under long daylight triggers the end of the vegetative and the start of the reproductive phase. The literature does not provide a clear indication of the exact photoperiod required to trigger the phase change, but it is evident that it must be greater than the period of darkness (14–16 h have been used to induce flowering in growth chambers) and last for a few weeks [33,34,40,41]. Typically, inflorescence stalks start to develop in mid to late spring [3,41,42].The base PlantainForage model uses the accumulation of days in which the photoperiod is greater than 11.5 h to trigger the end of the vegetative phase. Different cultivars may have different requirements, broadly classified as early or late flowering [41].

The reproductive phase is when the plants are actively growing reproductive organs (stalks, inflorescence, and seed). Flower stages and seeds development are not explicitly described in the current model, which defines only a generic ‘inflorescence’ organ. Reports indicate that seed ripening occurs approximately 8–10 weeks after flowering, with commercial cultivars closer to the low end of the range [3,13]. The length of the reproductive phase appears to be related to the onset of flowering [41]. However, flowering generally continues throughout the summer into autumn and seems to be curtailed only by frosts and/or short days [40]. In the model, photoperiod is used to define the end of the reproductive phase, following a similar approach used to start it. With the accumulation of short days (days with daylight <11.5 h) used to trigger the end of the reproductive phase. This approach also ensures a phenology reset even in warmer climates. The model also uses the variation of photoperiod throughout the year to control the partitioning of new biomass into various organs, as described in a later section.

### Morphology

The PlantainForage model considers five organs to describe the plant: leaves, stems (or stalks), inflorescence, taproot, and roots. As with other models built using PMF, each organ has a biomass component that holds the amount of carbon (C) and nitrogen (N) in three pools, structural, non-structural, and metabolic [28]. New biomass is allocated to each organ based on its relative demands, aiming to maintain specific biomass ratios between organs. These ratios can vary depending on phenology, with more biomass being allocated to above-ground organs (shoot) during the reproductive phase. The primary source of C biomass is photosynthesis, with some re-translocation from taproots also possible. The supply of N encompasses uptake from the soil and re-translocation from senescing organs.

**Fig. 3:**
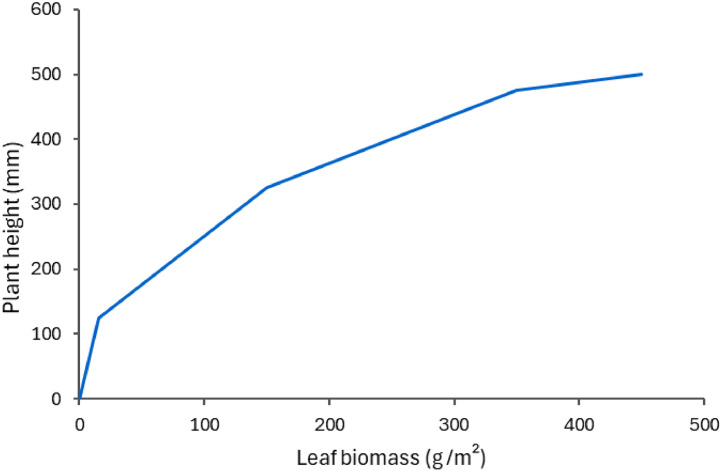
Plant height as function of leaf biomass used in the PlantainForage model, based on [21].

The canopy is basically composed of the Leaf organ. This sub-model has parameters to define the conversion of biomass into leaf area (specific leaf area) as well as plant height. The height and leaf area indices (for both live and dead material) are passed, along with the light interception coefficient, onto MicroClimate (see apsim.info documentation [43]). This is the model that calculates the actual light interception for plantain, and partitions the light resource among any other competing species in the sward. The MicroClimate model also computes water demand for plantain and again partitions the available water among the various species. The current model assumes a fixed specific leaf area (21 m^2^/kg) to convert biomass into leaf area [15,44,45], ignoring variations due to environmental conditions (e.g., light or N availability). A simple function derived from data collected in a New Zealand trial is used to estimate plant height from leaf development (Fig. 3) [21]. This links height with leaf biomass and ensures that plant height is reset whenever a defoliation event happens. Variations due to cultivar or environmental factors are not currently considered.

The Stem organ represents all the stems/stalks in the plant, without distinction between developmental stage. Stems only develop during the reproductive phase to support the inflorescence and have no specific function within the model. The Inflorescence organ accounts for all the reproductive parts of the plant (flowers, pods, and seed). The model does not distinguish between the age or placement of inflorescence in the canopy. Seeds are not explicitly simulated in the current version. Palatability of stalks and inflorescence is generally low and grazing animals will avoid them [19,20]. In APSIM, this distinction is not part of the plantain plant model. Any selective defoliation must be set up in the defoliation agent (e.g., animal models or manager scripts).

**Fig. 4:**
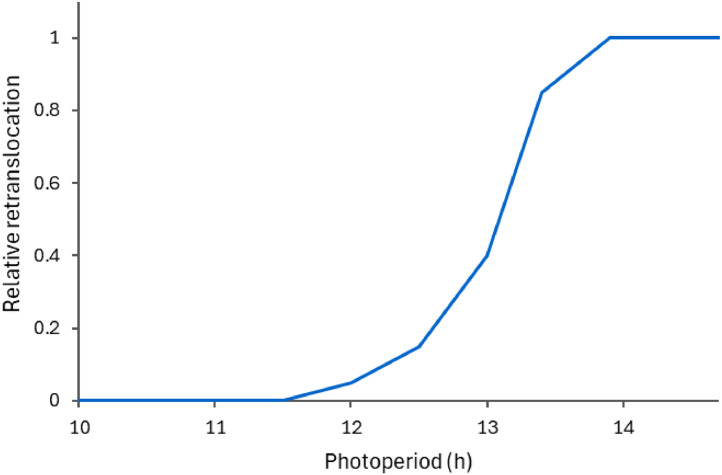
Factor describing the relative effect of photoperiod used in the PlantainForage model to modify re-translocation of biomass from taproots.

The Taproot organ represents the thick roots and crown. These are the primary reserve organs that can supply sugars and N to support shoot growth, especially early in spring and following defoliations. In the model, this is accomplished by assuming that taproots contain a sizeable fraction of non-structural biomass which can be made mobilised to support new growth via re-translocation. The amount of data available to parameterise how much reserves are stored in plantain taproots is limited. Published data suggest that sugars plus fructans make up 4–11 % of taproots’ biomass in plantain [46], compared to 15–35 % in chicory, which is consistent with plantain forage having much smaller taproots [15]. In the PlantainForage model, the parameters related to re-translocation (StructuralFraction, DMRetranslocationFactor, and NRetranslocationFactor) were estimated to reflect those findings (0.6, 0.1, and 0.1; respectively). However, these values should be updated when more data become available. Furthermore, an additional factor, a function of photoperiod (Fig. 4), is used to limit re-translocation to the period in which the plants are actively growing (spring to early autumn).

The Root organ represents all the fine roots of the plant. This organ is distributed over the soil profile, with specific demands and biomass partition for each soil layer in the simulation, down to the rooting depth. Most plantain roots are concentrated near the surface, but can extend down to a depth of one metre or more. The growth pattern also shows a lot of architectural variation [2,16,47,48]. The PlantainForage model distributes root biomass down to one metre, provided that soil conditions permit (setting the soil parameter ‘XF’ to zero will prevent roots growing in that layer [49]). A specific root length of 120 m/g is used to convert biomass into root length [38,48,50]. Water and N uptake is computed based on the distribution of roots in each soil layer. The uptake process is mediated by the SoilArbitrator model (see apsim.info documentation), which considers the demand from the species in the sward with the supply from the soil to define the actual amounts taken up by each species in the simulation (if there are more than one).

### Biomass accumulation and translocation

Biomass increases as a function of photosynthesis, which is simulated exclusively by the leaf organ in the PlantainForage model. Data for photosynthesis in forage plantain are very sparse and data from studies on wild varieties had to be used to support parameterisation. Reported values for CO_2_ assimilation rates in plantain are generally consistent [44,51,52] and follow a classic relationship with light intensity (directly proportional) as well with N concentration in the leaves (inversely proportional). Reported values of photosynthetic rate indicate that these are similar or slightly higher in plantain forage when compared to other leafy forages like chicory [51,52]. PMF uses radiation use efficiency (RUE) values to calculate the daily photosynthetic rate. Based on published data, the total RUE for plantain was estimated to be around 2.0 g DM/MJ [44,53].

**Fig. 5:**
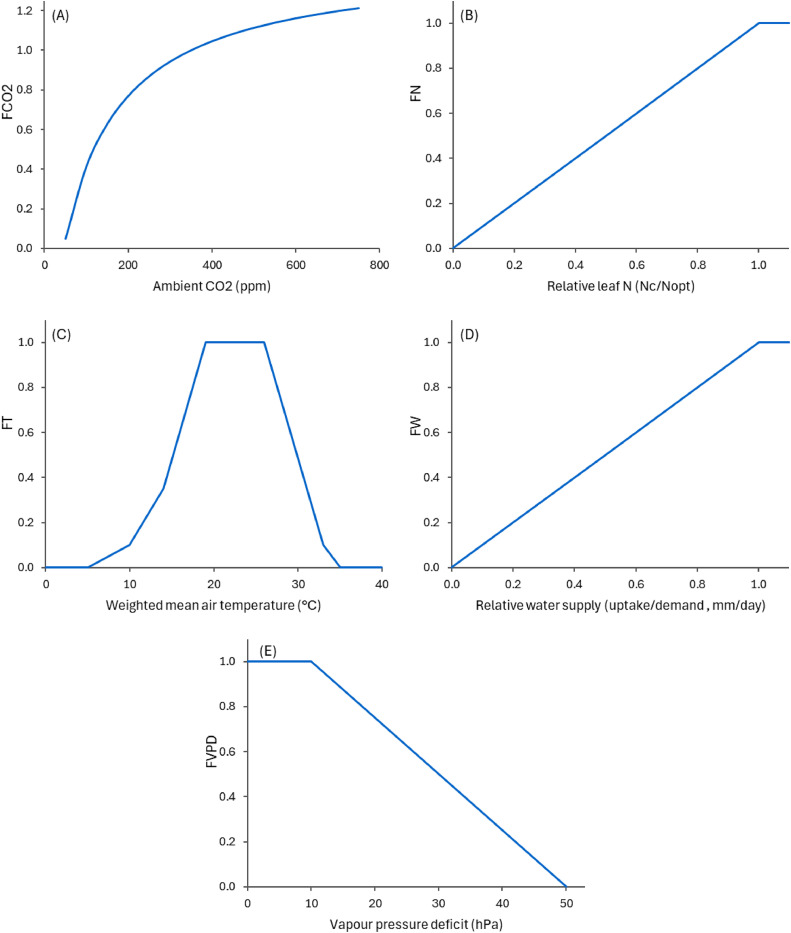
Relative responses of photosynthesis to several environmental factors in the PlantainForage model, where a value of one represents unrestricted growth, or at a reference value for the case of CO_2_ response. In (A) FCO2 is the response factor to atmospheric CO_2_ (reference at 350 ppm)[57]; in (B) FN is the response to N concentration in leaves (relative to optimum); in (C) FT is the response to the weighted mean air temperature ($0.75T_{max}+0.25T_{min}$); in (D) FW is the response to water supply, as a function of the ratio between demand and supply; and in (E) FVPD is the response factor to vapour pressure deficit.

**Fig. 6:**
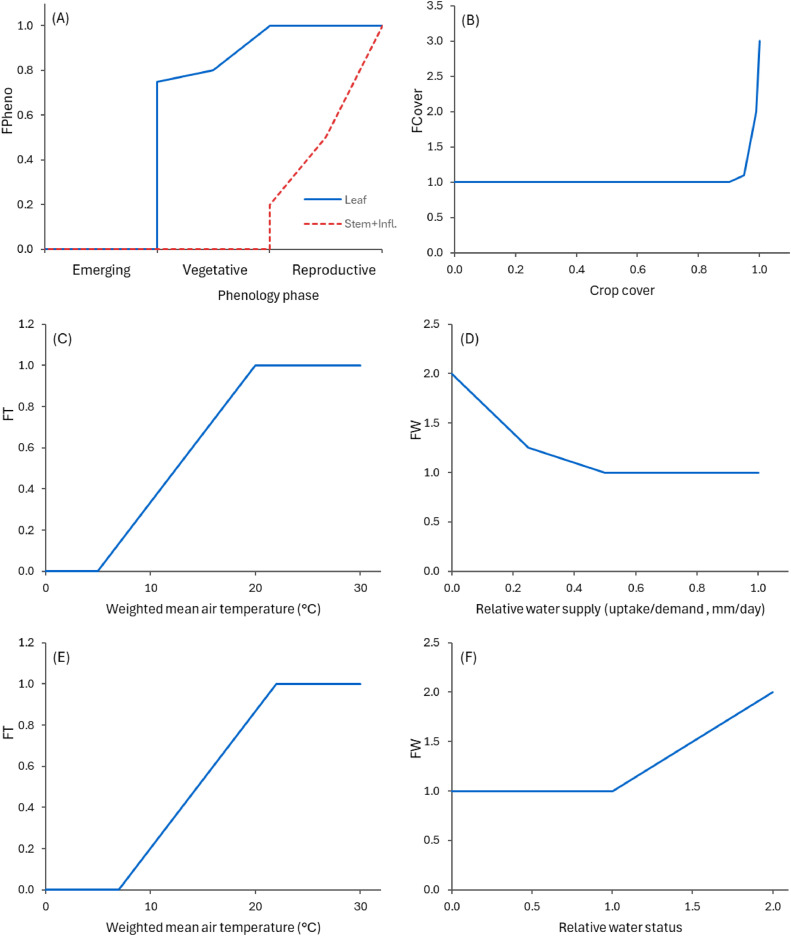
Effects of environmental factors on the senescence and turnover of plantain organs. These vary from zero (no turnover) and can go beyond one (at reference turnover rate). In (A) FPheno is the relative turnover rate as function of phenology state for leaves (solid line) and other above-ground organs (dashed line); in (B) FCover is the effect of crop cover on leaves; in (C) and (D) FT and FW are the effects of air temperature and soil water availability for above-ground organs; in (E) and (F) FT and FW are the effects of temperature and soil moisture status (where zero represents wilting point, one is field capacity and two means soil moisture at saturation) on the relative turnover of below-ground organs.

The current model considers that photosynthesis and biomass turnover are affected by several environmental factors, such as temperature and water availability. Due to limited information available specific to plantain, generic responses are used (Figs. 5 and 6). Plantain is ubiquitous in meadows in subtropical and temperate climates around the world, and it is generally considered to be tolerant to high temperatures, but not to cold [1,2,53,54]. The growth pattern of forage plantain can vary significantly among cultivars, with some being selected for winter growth and still outperforming temperate grasses in summer [2,20,35]. However, survivability in harsh winters in the USA was found to be low [40,54]. Forage plantain is also often mentioned as a drought-tolerant species [2,46,55], but less so than deep-rooted species such as chicory or red clover. The use of reserves from the taproots may be a key mechanism for plantain’s drought resistance, as is the case for many herbs [46,52]. Sensitivity to nitrogen concentration in leaves follows a simple function similar to other plants in APSIM, while for vapour pressure deficit (VPD), a simple function is adapted from a comprehensive review on plant responses to VPD [56].

The sparse available data suggests that plantain has a relatively slow senescence rate compared to other meadow herbs [58,59]. For the model, reference values were defined to reproduce the general pattern from observations, but this is an area that will need further investigation. The reference senescence rates (0.005 for leaf, 0.1 for stems, 0.25 for inflorescence, 0.005 for taproots, and 0.01 for roots) are adjusted using a phenology factor (Fig. 6A). This accounts for seasonal variations and growth stage, with older plants having a higher turnover rate. The rate is further adjusted according to environmental factors (Fig. 6), which are defined using generic responses to the stand condition (i.e. cover) and the environment. Biomass turnover is assumed to increase with cover, particularly at high levels, due to resource competition (currently, this is valid only within the PlantainForage model and will not account for inter-species competition). For environmental factors (temperature and soil moisture), general responses are also assumed, with variations for above- and below-ground organs. The model assumes that a large water deficit can accelerate senescence of leaves and other above-ground organs, but will have a negligible effect on below-ground senescence. On the other hand, excess moisture is considered to have a detrimental effect for below-ground organs, accelerating senescence, but above-ground organs are not affected. The sensitivity of plantain to conditions with either very low moisture or water logging is not clear, although the latter has been reported to be detrimental [60] (Plantago major seems to be more tolerant to water logging than *Plantago lanceolata* [1]). These responses require further verification and should be updated in the future as additional data become available.

**Fig. 7:**
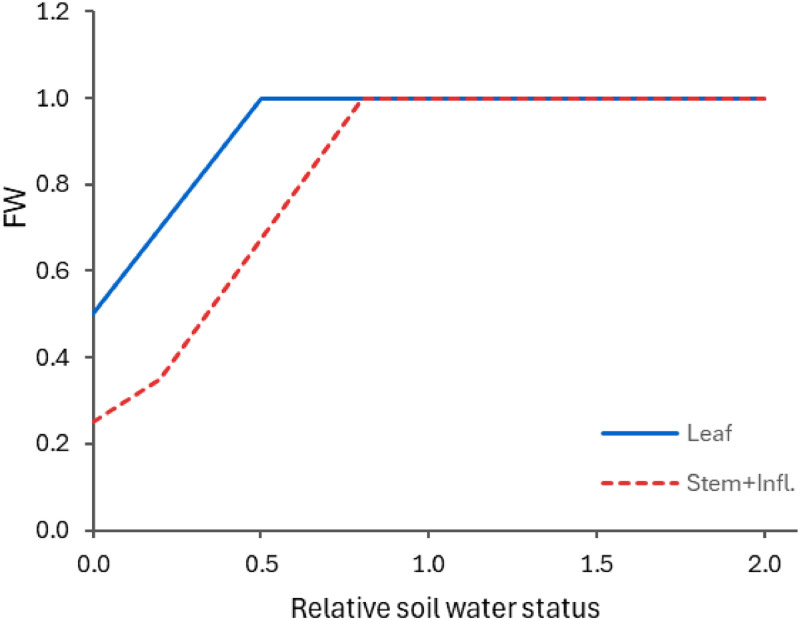
Effects of soil moisture status (where zero represents wilting point, one is field capacity and two means soil moisture at saturation) on the relative detachment rate for material of above-ground organs (solid line for leaves and dashed lines for the other above-ground organs).

It is worth noting that any below-ground biomass that senesces is immediately added to the soil organic matter pool, whereas above-ground organs retain dead material for a while and detach it into the surface organic matter pool over time. The detachment rate is modified by a factor that is a function of soil moisture (Fig. 7), which serves as a proxy to the overall system moisture. This implies that the detachment of plant material occurs slower in dry conditions due to the reduced biological activity.

### Biomass partitioning

**Fig. 8:**
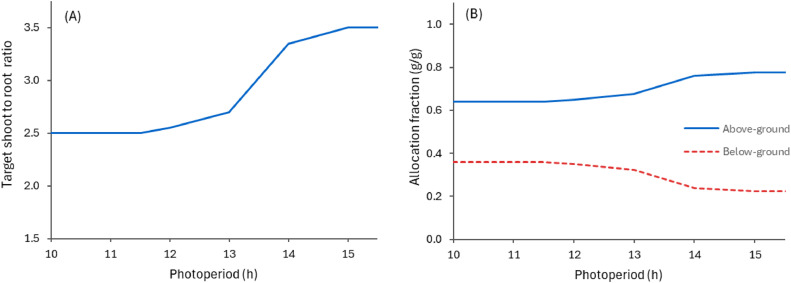
Variation in target shoot-to-root ratio used in the PlantainForage model during the reproductive phase to allocate new biomass into above- and below-ground organs (A). The actual allocation fractions change depending on the current biomass ratio (see Eq. (2)), the right-hand graph (B) shows an example when the current ratio is fixed at 3.5.

The allocation of new biomass (growth) to the various organs in the PlantainForage model is regulated using ratios between the biomass of various organs, or group of organs (e.g. Shoot:Roots, Stem:Leaf) [61,62]. This approach accounts for the plasticity in biomass allocation in plants, which are particularly important after defoliation when the biomass of some organs is mostly removed from the plant. In this case, the plant will prioritise the re-growth of some organs (e.g. leaves) at the expense of others (i.e. roots). This implies that the plants have 'target' ratios that they attempt to maintain, and these can vary with phenology stage and resource availability. The validity and mechanisms regulating biomass ratios is still debated by physiologists, but they are very useful for modelling purposes [61,63]. In the PlantainForage model, this approach takes into consideration the target ($r_{target}$) and the current ratio ($r_{current}$) to determine the proportion of new growth to be allocated to pairs of organs (or group or organs). To illustrate how biomass allocation is computed, the shoot-to-root ratio is used as an example here. The proportion of biomass to allocate above-ground organs, or shoot ($p_{Shoot}$), is computed using:

(2) $$p_{Shoot}=\frac{r_{SR,target}^{2}}{r_{SR,target}^{2}+r_{SR,current}}$$

where $r_{SR,target}$ is shoot-to-root ratio targeted by the model, its value start at 2.5 reaching a potential value of 3.5 during the reproductive phase (Fig. 8), and reverting late in autumn. Note that these values may vary in the future with implementation of new cultivars or other model adjusts. $r_{SR,current}$ represents the ratio of current biomass in above-ground organs (Leaf, Stem, and Inflorescence) to those below-ground (Taproot and Root).

**Fig. 9:**
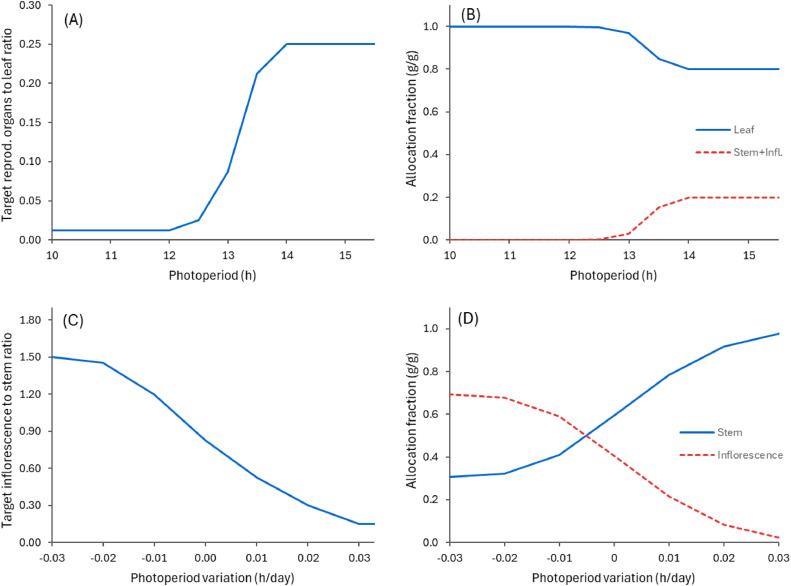
Target ratios of biomass between reproductive organs (Stem and Inflorescence) and Leaf (A), and between Inflorescence and Stem (C) that are used in the PlantainForage model during the reproductive phase to allocate new biomass into each above-ground organ. The actual allocation fractions change depending on the current biomass ratios (similar to Eq. (2)). The right-hand graphs show examples when this ratio is equal to 0.25 for reproductive organs and Leaf (B) and 1.0 for Inflorescence and Stem (D).

Further partitioning of newly grown biomass into each organ is similarly determined using ratios between Leaf and reproductive organs (Stem and Inflorescence), and then between Stem and Inflorescence (Fig. 9). Photoperiod and the variation in photoperiod over the season are then used to modulate the target ratios such that the model can capture the observed seasonal variation in biomass allocation. This simple approach ensures stem growth is prioritised early in the reproductive phase (when photoperiod is increasing) while flower and seed development are favoured later on (when photoperiod starts to decrease).

**Fig. 10:**
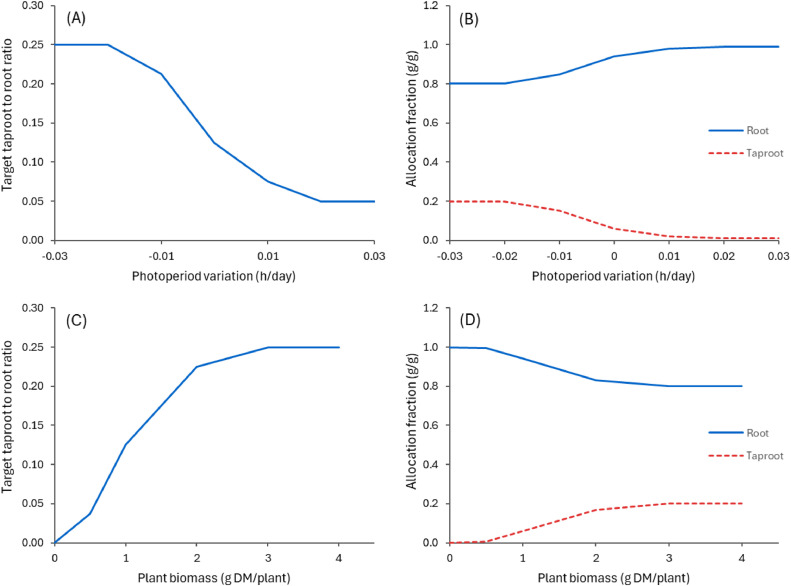
Variations in target biomass ratio between Taproot and Root used in the PlantainForage model to allocate new growth biomass into below-ground organs. The graphs show the relative variations as a function of photoperiod (A) and plant biomass (C). The allocation fractions used in the model are computed using the current biomass ratio (similar to Eq. (2)). The right-hand graph shows examples of relative allocation fractions as function of photoperiod (B) and plant biomass (D) when the current ratio is set to 0.25.

The allocation of biomass to below-ground organs also follows a similar approach, with the target ratio between Taproot and Root varying in response to changes in photoperiod. This ensures that allocation prioritises taproots (build up reserves) in autumn when the photoperiod decreases (Fig. 10). The ratio is further adjusted based on plant biomass (g DM per plant) providing a compromise method ensuring that root growth is prioritised in smaller, young, plants, with taproot development becoming more significant in larger, older plants.

### Nitrogen balance

The nitrogen content of the various plant organs is defined by three concentration thresholds (Table 1). These parameters were estimated from data collected during various trials covering different management regimes and cultivars [21,24,44,51,54,64]. Unfortunately, none of these datasets are comprehensive enough to provide definitive parameters for different varieties. Further work is needed, especially with newer cultivars and for below ground organs. In the model, plant growth is maximum when the N content of plant leaves is equal or above the critical value, and a penalty occurs if the content falls below that threshold (Fig. 5B). In conditions with high soil N, uptake can lead to the N concentration of plant organs exceeding the critical level, representing luxury uptake. This excess uptake can result in plants N content getting up to the maximum value. This luxury N content can be re-translocated and used by the plant to support growth when soil N is insufficient to meet demand. Upon senescence, the amount of nitrogen above the minimum concentration also becomes available for use during plant growth.

**Table 1:** Nitrogen (N) concentration thresholds for each organ in the PlantainForage model.

Organ	Minimum N (g/g)	Critical N (g/g)	Maximum N (g/g)
Leaf	0.023	0.035	0.050
Stem	0.005	0.025	0.025
Inflorescence	0.025	0.035	0.035
Taproot	0.005	0.020	0.020
Roots	0.005	0.015	0.015

### Defoliation

The removal of crop biomass in the PlantainForage model follows the underlying processes in PMF. Biomass (C and N) can be removed from any organ using the RemoveBiomass command. This can be called from anyone of the auxiliary models that are available in the APSIM framework (Operations, SimpleGrazing, or Manager scripts – see apsim.info). From the PlantainForage model perspective, any defoliation is treated equally, i.e., there are no differences if the sward is mown or grazed by animals. Some variations can and often are implemented by additional actions triggered in parallel to a defoliation, such as the return of defoliated material as residues or as dung and urine. The current model assumes that phenology is not affected by defoliation, which seems to be the case in most situations in grazing systems (perhaps with the exception of very heavy defoliations). Nonetheless, PMF does allow setting the phenology stage from another sub-model in APSIM, which can be used in simulations where this is deemed necessary.

## Method validation

### Field trials

Validation simulations were set up in APSIM based on several field trials performed at different locations across New Zealand. These cover different climatic and edaphic conditions as well as different management. Generally, the trials have been managed as cut and carry, with only one under grazing. The management in those trials encompasses a wide range of N fertiliser applications, the presence or absence of irrigation, and variable defoliation pressure. Further details including the actual dates and amounts for irrigation, fertiliser applications, and defoliation can be found in the simulation files (available at github.com/APSIMInitiative/ApsimX/Tests/Validation/PlantainForage). A brief description of each trial is given below.

**FRNL-Lincoln**. Trial conducted at Lincoln University Research Dairy Farm (43° 38′S, 172° 26′E), between 2014 and 2016, as part of the Forages for Reduced Nitrogen Leaching programme [65]. Plots with pure plantain stands were sown in autumn 2014 and involved six N fertiliser treatments (nominal N rates of 0, 50, 100, 200, 350 and 500 kg/ha/yr) with four replicates. The plots were irrigated and mown regularly; measurements comprised biomass yield and quality indicators (here N content is used). The site has an imperfectly-drained Pallic soil and a dry temperate climate (annual rainfall of 650 mm and daily average temperature of 12.1 °C).
**FRNL-Ruakura**. This field trials was performed at DairyNZ's Scott Farm, in Ruakura (37° 46′S, 175° 22′E), between the spring of 2014 and 2016 [66]. This was also part of the Forages for Reduced Nitrogen Leaching programme and had the same treatments (N rates of 0, 50, 100, 200, 350 and 500 kg/ha/yr), with three replicates, and broadly similar management to the one in Lincoln, except that it was not irrigated. Biomass yield and mean N content of harvested material were recorded at each defoliation (also done by mowing). The site has a well-drained Allophanic soil and a wet temperate climate (annual rainfall of 1190 mm and daily average temperature of 13.7 °C).
**ScottFarmFD902**. This was a defoliation trial conducted at Scott farm, Ruakura, from 2010 to 2012 by DairyNZ [21]. The study investigated the effect of cut height (which defined the rotation length) and residual height on plantain growth. Defoliations were triggered when the sward reached target heights of 150, 250, 350, and 450 mm, and the crop was cut down to heights of either 40 or 70 mm (±10 mm). Irrigation was applied in the first year only and some N fertiliser was applied over summer. Data reported from the trial include biomass yield, botanical composition (presence of weeds), and leaf size. Light interception was also measured in this trial.
**LincolnRDF**. Field trial performed at Lincoln University Research Dairy Farm, between 2010 and 2012. The experiment had two nitrogen treatments, a low (at 125 kg N/ha/yr in five applications) and a high (at 250 kg N/ha/yr) rate, applied with the same number of split applications. Yield was determined in quadrats mown down to 45 mm every 4 weeks. After this, animals were allowed to graze the areas, aiming for a residual height of to 45 mm. This was followed by further mowing if necessary to tidy up the plots. When grazing dates coincided with the allotted N schedule, urea was then applied by hand within seven days following the grazing.
**MasseyDairyNo1**. Part of a field trial performed at No1 Dairy Unit at Massey University in Palmerston North, New Zealand (40° 22′S, 175° 36′E) [67]. The experiment was conducted during 2011–2012 (first growing season) and 2012–2013 (second growing season). Three pasture treatments were evaluated: (i) chicory, (ii) plantain, and (iii) an herb-clover mix pasture, under two grazing frequencies; every two or four weeks. Perennial ryegrass/white clover pasture was also established and grazed to provide a benchmark. Only the data from pure plantain plots were used here. The site has a well-drained sandy soil and a temperate climate (annual rainfall of 970 mm and daily average temperature of 13.3 °C).

### Ancillary data and validation metrics

For each of the trials, the soil type was known and a combination of published data as well as data from the New Zealand National Soil Database (ManaakiWhenua-LandcareResearch) was employed to derive the parameters needed for soil models in APSIM [49]. Daily weather data were obtained from nearby (<3 km away) weather stations (Cliflo, NIWA).

The model performance was analysed using a series of goodness-of-fit measures, as is standard in APSIM validations (see apsim.info). These include the coefficient of determination (R²), the Nash-Sutcliffe model efficiency coefficient (NSE), and the root mean square error (RMSE) [68]. Graphical comparisons between predicted and observed values are also summarised in the figures below.

### Model performance

**Fig. 11:**
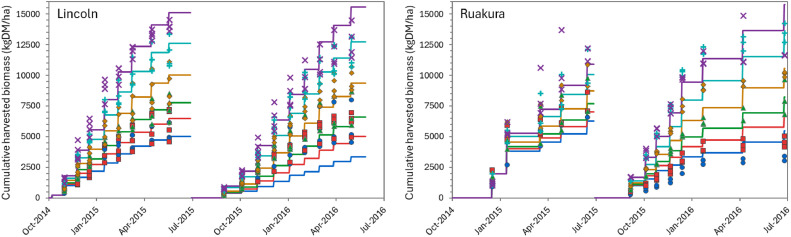
Predicted (lines) and observed (dots) values for cumulative biomass yield over two growing seasons for the FRNL trials, at Lincoln and Ruakura. Treatments consisted of annual N fertiliser rate at 0 (●), 50 (■), 100 (▲), 200 (◆), 350 (+), and 500 kg/ha (×).

The overall performance of the model can be considered “very good” for simulating biomass production over the growing season (Table 2), according to APSIM standards (NSE > 0.8). Predictions for individual defoliation events were less accurate, but still generally “satisfactory” (NSE > 0.5). There was considerable variation in performance between datasets. For the two FRNL trials (see also Figs. 11, 13, and 14) and the FD902 (see Fig. 15), which consisted of small plots under well-controlled settings, the model is capable of describing biomass production very well, including variation within the season. As expected, the performance was not as good for the less controlled and/or grazed trials when comparing within-season yields, for which there are more factors contributing to the variability (e.g. grazing preference and urine patches), although cumulative values were still good (Table 2 and Figs. 16 and 17). It is important to note that the trials have not been designed to be used for model comparison specifically and some of the measurements may not align well with the assumptions made on the simulations. For instance, it is assumed in the model that a fixed proportion of any existing above-ground organ is harvested on each defoliation, but there is no record of the proportion of various plant parts actually harvested, nor whether ‘weeds’ had always been removed or were analysed together with plantain. Grazing and cut residuals can be quite variable in terms of biomass, especially when only approximate height is recorded. These aspects add noise to the data and thus make comparisons on specific dates much more difficult.

**Table 2:** Summary of some measures for the performance of the PlantainForage model when compared with observed data from five New Zealand field trials. Where: harvested biomass represents the amount harvested on each defoliation event, for each replicate; cumulative biomass yield is the harvested biomass accumulated over one growing season (1 July to 30 June); and cumulative N yield is the amount of N in the harvested material accumulated over the season.

Dataset	Harvested biomass (kg DM/ha)			Cumulative biomass yield (kg DM/ha)			Cumulative N yield (kg N/ha)		
	R²	NSE	RMSE	R²	NSE	RMSE	R²	NSE	RMSE
FRNL-Lincoln	0.50	0.47	449	0.90	0.89	1204	0.89	0.85	35.5
FRNL-Ruakura	0.45	0.44	759	0.89	0.89	1187	0.90	0.87	33.6
ScottFarmFD902	0.65	0.63	668	0.79	0.73	1971	-	-	-
LincolnRDF	0	−1.39	591	0.87	0.87	939	0.75	0.73	33.6
MasseyDairyNo1	0.13	0.11	929	0.73	0.68	2618	-	-	-
All data	0.53	0.51	417	0.82	0.80	1814	0.87	0.85	35.0

Above-ground biomass accumulation between defoliation was only recorded in the FRNL trials, and more extensively only for the Lincoln site. The model performed reasonably well, with goodness-of-fit measures in line with those of within-season defoliation amounts (R² was 0.57, NSE was 0.54, and RMSE was 328 kg DM/ha for Lincoln; and 0.50, 0.48, and 429 kg DM/ha for Ruakura, respectively). As was observed with defoliated biomass amounts, the performance tended to be worse in the second year of the trial (example in Fig. 11). This is likely an indication of the model’s current inability to account for a decline in plant population, or the fact that weeds were not considered in the current simulations.

**Fig. 12:**
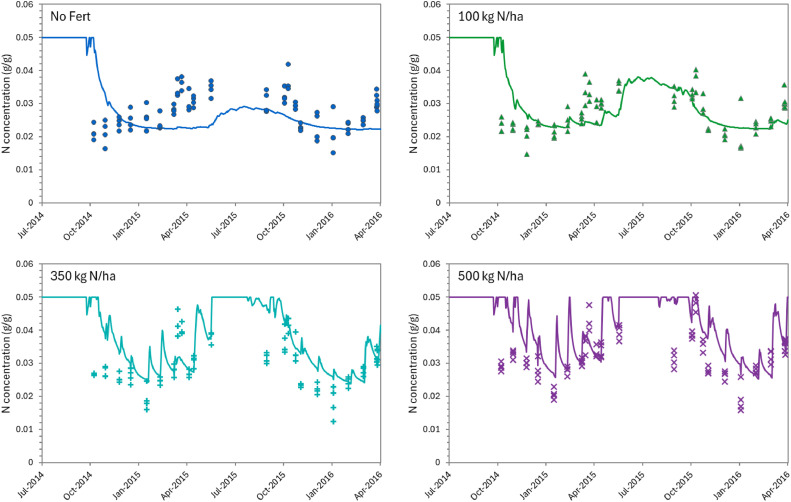
Predicted (lines) and observed (dots) seasonal variations in N concentration two growing seasons for the FRNL trial at Lincoln. Four treatments are show, for annual N fertiliser rates of 0 (●),100 (▲), 350 (+), and 500 kg/ha (×).

**Fig. 13:**
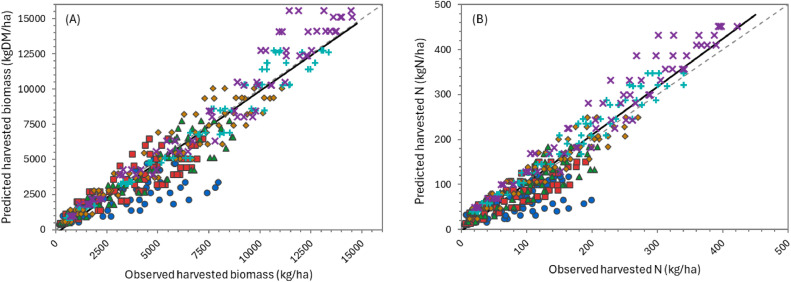
Predicted versus observed values for cumulative harvested biomass (A) and N yield (B) for the FRNL-Lincoln trial. Treatments were annual N fertiliser rate at 0 (●), 50 (■), 100 (▲), 200 (◆), 350 (+), and 500 kg/ha (×). The solid line represents the linear regression between predicted and observed values while the dashed line is the 1:1 relationship.

**Fig. 14:**
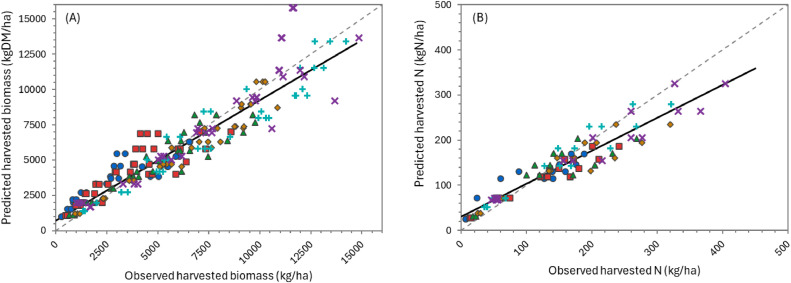
Predicted versus observed values for cumulative harvested biomass (A) and N yield (B) for the FRNL-Ruakura trial. Treatments were annual N fertiliser rate at 0 (●), 50 (■), 100 (▲), 200 (◆), 350 (+), and 500 kg/ha (×).The solid line represents the linear regression between predicted and observed values while the dashed line is the 1:1 relationship.

Only a limited amount of data were available to validate the dynamics of N in the plantain biomass. These consisted of the overall N content from defoliated material for some of the datasets. The model is capable of describing the seasonal pattern reasonably well (e.g. Fig. 12) and the overall amount of N harvested per season (Table 2 and Figs. 13, 14, and 16). However, considerable deviation still exists between values for individual defoliation events, indicating that this is an area that can be further improved after the collection of more data. Note that the measurements show large variability between replicates, suggesting large spatial variations and/or the need to improve the measurement methodology.

**Fig. 15:**
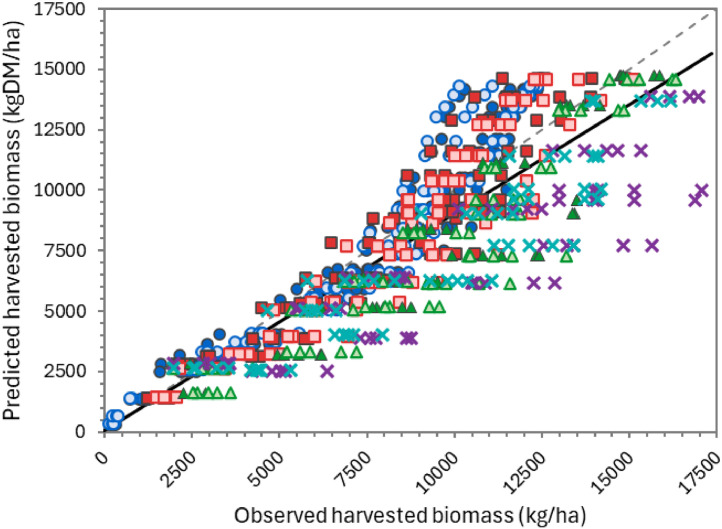
Predicted versus observed values for cumulative harvested biomass for the Scott Farm FD902 trial. Treatments consisted of varying cutting heights (trigger - residual), at 15–4 cm (●), 15–7 cm (○), 25–4 cm (■), 25–7 cm (⬜), 35–4 cm (▲), 35–7 cm (△), 45–4 cm (×), and 45–7 cm (×).The solid line represents the linear regression between predicted and observed values while the dashed line is the 1:1 relationship.

**Fig. 16:**
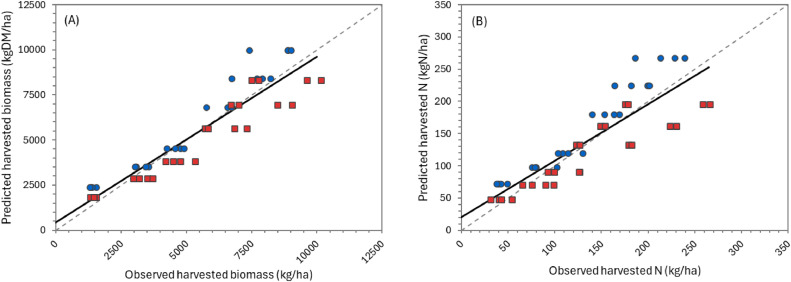
Predicted versus observed values for cumulative harvested biomass (A) and N yield (B) for the LincolnRDF trial. Treatments were two N fertiliser rates, a low (●) and a high (■). See text for details. The solid line represents the linear regression between predicted and observed values while the dashed line is the 1:1 relationship.

**Fig. 17:**
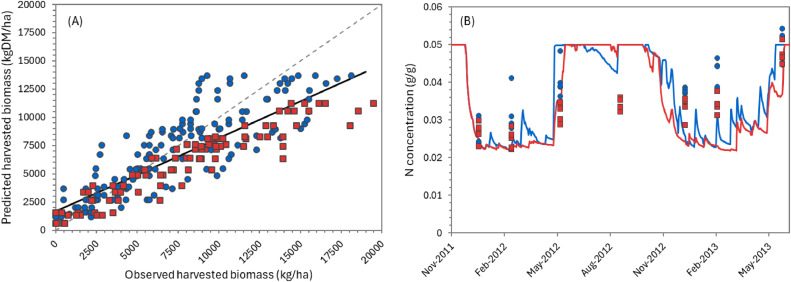
Predicted versus observed values for cumulative harvested biomass yield (A) and seasonal variation of N concentration in the herbage harvested (B) for the MasseyDairyNo1 trial. Treatments consisted of two grazing frequencies, every two (●) or four weeks (■). In (A), the solid line represents the linear regression between predicted and observed values while the dashed line is the 1:1 relationship. In (B), the solid line represents predicted values.

## Final remarks and areas for future improvements

The model described here is the second iteration of development initially started in 2019. By using APSIM, we were able to focus on the development of the plant model and can now use the full framework to explore interactions between plantain and the environment, particularly soil processes, as well as investigate the effects of management practices on plantain growth and on those interactions with the environment. Model performance varied from very good to satisfactory when predicting biomass and nitrogen harvested over a growing season. However, the performance was only satisfactory or poor when comparing against data at individual harvests. The model showed good sensitivity to treatments studied, especially N fertiliser rates. These results suggest the model can be used to predict annual biomass production for forage plantain with confidence, but predictions within a season must be taken with some caution. The calibration and validation datasets were mostly from New Zealand, some important environmental factors included temperature and photoperiod were in a limited range. Therefore, future validation is needed when applying the PlantainForage model in locations outside of New Zealand. Although a number of studies with plantain have been conducted over the last few years, the amount of data for specific model parameterisation remains limited. This is particularly acute for parameterising the allocation and re-translocation of biomass and nitrogen among the various organs. Lack of data on roots growth and distribution in the soil profile is also a major gap. This is important to improve model performance to estimate growth under different conditions (e.g., water stress) and regrowth after defoliation. Nitrogen balance and feed quality are also related to how biomass is allocated across different organs over the growing season, the data available are not yet detailed enough to parameterise the model with great confidence. Understanding the allocation of biomass to roots and its reallocation to support growth in spring and after defoliation is important to help simulate the persistence of plantain and guide plantain management practice on farm. Little is known about the rooting depth and the distribution of roots in the soil for New Zealand conditions. Similarly, more information is needed to discern variations between cultivars, for phenology, biomass allocation, and root distribution. This dearth of data makes it difficult to parameterise and evaluate the model’s performance on different environmental conditions and increases the uncertainty within simulations of mixed swards. Data for plantain is being slowly generated and will allow for future upgrades to the model, but we specifically recommend controlled experiments to assess biomass allocation and the impact of defoliation on pasture persistence, especially in multi-species swards. This is particularly important as some of the effects of plantain on N losses, a major selling point for the use of plantain in grazed swards, are linked to maintaining a minimum level of the crop in the field [23]. The PlantainForage model in its current form can be used to study the conditions needed and potential companion species, but more data are needed to ascertain its predictions and help to understand the relative advantages and disadvantages of plantain within farming systems. Nonetheless, the current model can contribute to the efforts investigating alternative crops and improving management of forage and livestock systems, reducing its environmental impact while maintaining productivity.

## Ethics statements

None are applicable to this work.

## CRediT authorship contribution statement

**Rogerio Cichota:** Conceptualization, Methodology, Software, Validation, Writing – original draft, Writing – review & editing, Funding acquisition. **Xiumei Yang:** Data curation, Validation, Writing – review & editing.

## Declaration of competing interest

The authors declare that they have no known competing financial interests or personal relationships that could have appeared to influence the work reported in this paper.

## Data Availability

Data will be made available on request.
